# Healthcare consumption among subjects with otitis media undergoing middle ear surgery—analysis of cost drivers

**DOI:** 10.1007/s00405-022-07483-8

**Published:** 2022-06-22

**Authors:** Aaran T. Lewis, Douglas Backous, Byung Yoon Choi, Rafael Jaramillo, Kelvin Kong, Thomas Lenarz, Jaydip Ray, Alok Thakar, Krister Järbrink, Myrthe K. S. Hol

**Affiliations:** 1 Cochlear Limited, Mölnlycke, Sweden; 2 Swedish Medical Center, Seattle, USA; 3 Bundang Hospital, Seoul National University, Seongnam, South Korea; 4 Caldas Hospital SES, Manizales, Caldas Colombia; 5 Hunter ENT, New Lambton Heights, Australia; 6 Medical University of Hannover, Hannover, Germany; 7 ENT Department, Sheffield Teaching Hospitals, Sheffield, UK; 8 All India Institute of Medical Sciences, New Delhi, India; 9 Department of Otorhinolaryngology, Donders Centre for Neurosciences, Radboud University Medical Center, Nijmegen, The Netherlands; 10 Department of Otorhinolaryngology/Head and Neck Surgery, University of Groningen, Groningen, The Netherlands

**Keywords:** Otitis media, Cholesteatoma, Middle ear surgery, Economic burden, Cost analysis, Audiological care, Healthcare costs, Hearing loss

## Abstract

**Purpose:**

To map healthcare utilized by subjects with chronic otitis media, with or without cholesteatoma and perform a cost analysis to determine key drivers of healthcare expenditure.

**Methods:**

A registry study of 656 adult subjects with chronic otitis media that underwent a middle ear surgery between 2014 and 2018. Healthcare contacts related to all publicly funded specialist ENT care, audiological care and primary care for a disease of the ear and mastoid process were extracted. The data are extracted from the Swedish National Patient Registry on subjects that reside in western Sweden.

**Results:**

Subjects made 13,782 healthcare contacts at a total cost 61.1 million SEK (6.0 million EUR) between 2014 and 2018. The mean cost per subject was 93,075 SEK (9071 EUR) and ranged between 3971 SEK (387 EUR) and 468,711 SEK (45,683 EUR) per individual. In the most expensive quartile of subjects, mean cost was 192,353 SEK (18,747 EUR) over the 5-year period. These subjects made 3227 ENT contacts (roughly four each year) and 60% of total costs were associated with in-patient ENT care.

**Conclusion:**

Patients with chronic otitis media are associated with high ENT resource utilization that does not diminish after surgical intervention and the disease places a long-term burden on healthcare systems. Significant costs are attributed to revision surgeries, indicating that these patients could be managed more effectively. In many such cases, reoperation cannot be avoided, especially due to recurrence of cholesteatoma. However, in some patients, when the indication for subsequent surgery is only hearing improvement, alternative options, such as hearing aids or implants, should also be considered. This is especially true in difficult cases, where revision ossiculoplasty is likely.

## Introduction

Chronic otitis media (COM) is a common issue encountered in ENT practice that can lead to hearing loss by damaging the middle and inner ear [1, 2]. In developing countries, COM is the most common cause of hearing loss [3]. The focus of treatment is to achieve a safe and dry middle ear space and restore maximum hearing potential, which is typically achieved through an array of surgical interventions, including mastoidectomies and tympanoplasties.

COM is a heterogeneous disease and the level of care and the complexity of surgical interventions required can vary substantially depending on the severity of infection and the status of the middle ear space in individual patients. Advanced surgeries are more commonly required when there is presence of cholesteatoma or when there is involvement of the middle ear ossicles [4, 5]. Furthermore, revision surgeries may be required in cases where infections persist, where recurrent or residual cholesteatoma is detected or suspicious, or to correct a failed ossicular reconstruction [6]. Cases requiring multiple surgeries are likely to impart significant time and financial burden on the healthcare system [7] and are also associated with risks and complications [8]. It is estimated that around 30% of patients do not receive a substantial hearing improvement following middle ear surgery [9].

The main objective of this study is to estimate the healthcare burden of patients with COM undergoing middle-ear surgery and to study how patterns of healthcare consumption may vary over time. Specific aims were to calculate the consumption of ear, nose and throat (ENT) healthcare and audiological care in order to identify explanatory factors for excess costs and lengthy episodes of care. This mapping exercise aims to provide a better understanding of the disease by quantifying direct healthcare costs and to identify factors that are of importance from a health economic perspective.

## Materials and methods

### Study design and study population

The data on healthcare consumption was extracted from a healthcare database covering information on all publicly funded healthcare for 1.7 million inhabitants of western Sweden (Region Västra Götaland). Sweden provides a tax-funded healthcare system that covers the entire population and only a small percentage of total healthcare services are financed privately. The contribution from patient fees is equivalent to around 4% of the total net cost of public healthcare in Sweden [10]. Region Västra Götaland is the second most populous region in Sweden and is representative of the general population in terms of demographics, socioeconomic factors and healthcare consumption [11, 12].

The sample of patients for which the data on healthcare is extracted were 18 years of age or older, received a diagnosis of chronic otitis media, with or without cholesteatoma (ICD-10, H65-H67) [13] during 2014–2018 and underwent a middle ear surgery during the same years. The data on healthcare utilization are extracted for the same years and presented by patient-year, providing a 5-year window into the registry. Middle ear surgeries were defined as any of the following (Nordic Medico-Statistical Committee [14]); Exploration of middle ear (DCA30), Myringoplasty (DCD00), Tympanoplasty (DCD10), Excision of lesion of middle ear (DCB00), Other operation on eardrum and middle ear (DCW99), Plastic repair of ossicular chain (DDD05), Cortical mastoidectomy (DEB00), Attico-antro-mastoidectomy (DEB10), Attico-antro-mastoidectomy and tympanotomy (DEB20), Attico-antro-mastoidectomy and tympanoplasty (DEB25), Radical mastoidectomy (DEB30).

### Data and healthcare costing

The data coverage included all care contacts made within and outside of the region between 2014 and 2018 to publicly funded specialist ENT care, audiological care and primary care with a diagnosis for a disease of the ear and mastoid process (ICD-10-CM codes H60-H95) [13]. Extracted data covered information regarding healthcare contacts, including the patient’s age and sex, date for contact, level of care, professional visited (outpatient care), diagnosis/diagnoses, diagnosis-related group (DRG) and cost for contact (specialist ENT care) and when applicable, code for surgery and number of bed days. Individual subjects were assigned a unique identifier and all contacts were linked to this identifier to enable the creation of episodes of care made by the same individual over the 5-year period.

Costs for visits to audiological care and primary care were based, when not specified in the data, on the price for a visit made by an individual not being a citizen in the region [15]. Specialist ENT care in the region was assessed using standardized costs in accordance with the regional compensation model for each year of consumption, as specified in the data extracted from the registry and inflated to the price of 2018 using the health sector price index [16]. Publicly funded private care and care outside the region was assessed using the actual compensation paid to providers. All costs were measured and analyzed in Swedish kronor (SEK). Indicative costs in Euros are presented using the average conversion rate for 2018 (1 EUR–10.26 SEK) [17].

### Statistics

Descriptive statistics are reported with mean and standard deviation (SD) for continuous variables, unless stated otherwise. Kolmogorov–Smirnov and Shapiro–Wilk test were used to assess the normality of continuous variables. All statistical analysis was performed using IBM SPSS Statistics, build 1.0.0.1174, 64-bit edition (Armonk, NY, USA).

### Ethical approval

Ethical approval for the study was received from the Swedish Ethical Review Authority (Dnr: 2019-04498).

## Results

In total, 656 patients met the inclusion criteria. The average age was 45.6 years and 52% were females. They had made 13,782 healthcare contacts between 2014 and 2018 for an ENT-related issue. On average, individuals in this group made 21.0 contacts (range 2–161; SD 14.4) during the 5-year period. Of these contacts were 56.2% made to specialist ENT care of which 2.4% (*n* = 332) were in-patient stays, 26.0% (*n* = 3583) were made to audiological care and 17.8% (*n* = 2447) were made to primary care with a diagnosis for a disease of the ear and mastoid process.

**Fig. 1:**
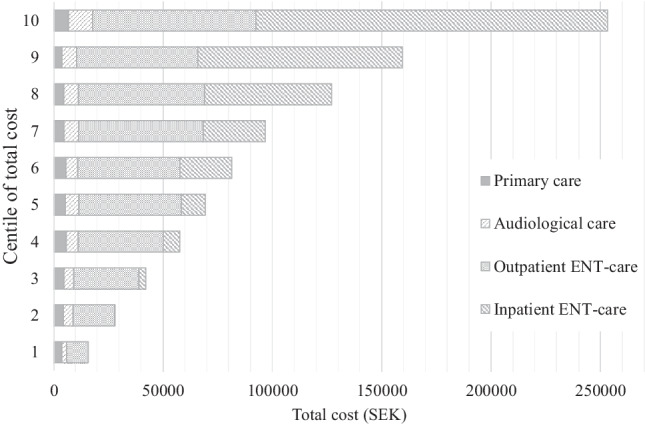
Centiles of total cost for all patients by category of care in SEK

The total cost was 61.1 million SEK (6.0 million EUR) resulting in a cost per individual of 93,075 SEK (9,071 EUR) during the 5-year period and an average cost per contact of 4,430 SEK (432 EUR). Total costs varied hugely between individuals, from 3971 SEK (387 EUR) to 468,711 SEK (45,683 EUR). Average cost per individual in centiles of total cost can be seen in Fig. 1. The costs for primary care and audiological care are evenly distributed across centiles, except for those that are least and most expensive. The distribution of costs for outpatient specialist ENT care were indiscriminately distributed across all centiles, but the greatest costs were captured in the ten percent of subjects that are most costly. The cost for inpatient ENT care increases over time and represents a significant proportion of the total costs, particularly amongst the 10% of patients that represent the highest total cost.

With the distribution of costs in mind, focus was placed upon the 25% (*n* = 164) of patients that represent the highest cost. The most expensive cohort possess the greatest potential to benefit from a change in care as the target population for alternative interventions is not the primary care group, but patients that most frequently require hospitalization. These patients represent 51.7% of the total cost. This high-cost group were slightly older (46.4 years of age) and 48.8% of them were women. On average, this group made 31.4 contacts (range 4–161; SD 20.4) during the 5-year period with an average cost per contact of 6,120 SEK (597 EUR). Additional details on this group of patients are presented in Table 1. The mean cost per individual was 192,353 SEK (18,747 EUR) over the 5-year period and 60% consisted of costs for inpatient ENT care and 33% for outpatient ENT care. 183 inpatient care episodes covering 386 bed days were reported. Costs for primary care and audiological care represented only 7.0% of the total cost. A breakdown of specialist ENT care contacts is presented in Table 2. The 164 patients made 3,227 ENT contacts during the five years implying nearly 4 contacts with ENT care per person and year. Of these contacts, 245 were surgeries that represented 69.8% of the total cost for ENT care. It should be noted that as data are extracted over a 5-year period, complete episodes of care are not captured, still, there is a clear tendency of a medical issue being raised with 41% of these patients having their first contact with primary care. This also continues for some time with 23.2% of the patients making their first four contacts with primary care during the 5-year period.

**Table 1:** Distribution and healthcare costs by category of care among the costliest patient quartile

Healthcare service utilisation	% users	Healthcare contacts		Healthcare costs (SEK)	
		Mean ± SD	Median (min–max)	Mean ± SD	Median (min–max)
Primary care	69.5	3.9 ± 5.6	2 (0–41)	4956 ± 6942	3188 (0–52,138)
Audiological care	97.0	7.8 ± 11.1	6 (0–124)	8442 ± 14,911	6090 (0–177,601)
Outpatient ENT care	99.4	18.5 ± 14.0	15 (0–141)	63,513 ± 50,586	44,674 (0–382,631)
Inpatient ENT care	86.6	1.2 ± 0.7	1 (0–4)	114,593 ± 70,540	115,839 (0–401,216)
Total	100.0	31.4 ± 20.4	26 (4–161)	192,353 ± 64,964	171,610 (127,701–468,711)

**Table 2:** Healthcare costs for ENT specialist care by category of contact among the costliest patient quartile

	Number of contacts	Average number of contacts	Average cost (SEK)	Proportion of total cost (%)
Outpatient care				
Visits for diseases of the ears, nose, mouth and throat	1662	10.13	27,333	43.0
Operations on the ears, nose, mouth and throat	73	0.45	13,674	21.5
Visits for other problems	230	1.40	3369	5.3
Visits for upper respiratory and ear infections	433	2.64	7104	11.2
Ear bone surgery	14	0.09	2735	4.3
Endoscopy	43	0.26	844	1.3
Visit for biopsies	11	0.07	287	0.5
Visit to nurse	21	0.13	219	0.3
Skin ulcer, rescheduling or other treatment	16	0.10	212	0.3
Physician visits to other bone diseases	7	0.04	179	0.3
Physician visit for balance problems, dizziness	11	0.07	174	0.3
Visits for diseases of the skin and subcutaneous tissue	11	0.07	168	0.3
Others/not defined	490	2.99	7085	11.2
Visits due to postoperative and post-traumatic infections	9	0.05	131	0.2
Total	3031	18.48	63,513	100.0
Inpatient care				
Operations on the ears, nose, mouth and throat	117	0.72	82,200	72
Ear bone surgery	37	0.23	21,464	18,6
Other surgeries for skin and subcutaneous diseases	1	0.01	1304	1.1
Plastic surgery on skin and subcutaneous tissue	1	0.01	1257	1.1
Other major operations on the head or neck	1	0.01	1198	1.0
Other diseases of the ear, nose, mouth & throat	5	0.03	1621	1.4
Balance problems, dizziness	7	0.04	961	0.8
Upper respiratory and ear infections	6	0.04	783	0.7
Operations in infectious diseases	1	0.01	614	0.5
Postoperative & post-traumatic infections	3	0.02	571	0.5
Treatment complications without surgical procedure	3	0.02	439	0.4
Others/not defined	14	0.09	2720	2.4
Total	196	1.20	115,691	100.0

**Fig. 2:**
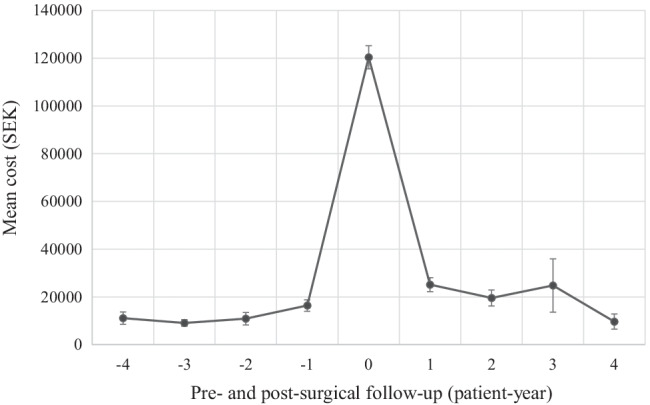
Mean yearly cost in SEK for the years before and after first-reported middle ear surgery (time point 0) for the costliest patient quartile. Error bars represent the standard error of the mean

**Fig. 3:**
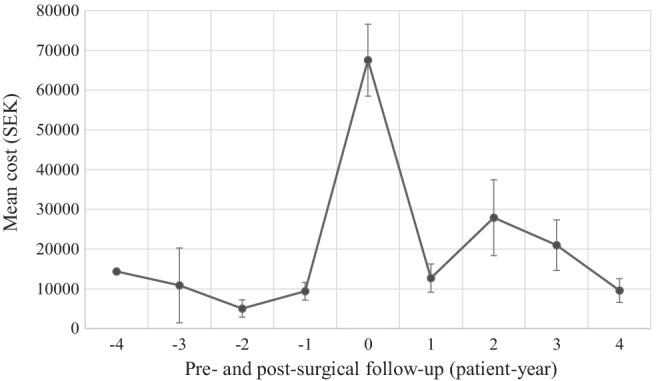
Mean yearly cost in SEK for the years before and after ossiculoplasty (time point 0) for all patients that underwent ossiculoplasty. Error bars represent the standard error of the mean

The most commonly captured surgery codes were C13O (operation on middle ear ossicles and other bony structures of the middle ear) and V15O (outpatient operation on ears, nose, mouth or throat), which were recorded for 29 and 412 patients, respectively. In total there were 33 procedures carried out under C13O at a mean cost of 30,577 ± 20,955 SEK (2980 ± 2042 EUR) per contact. The mean cost of patients requiring this procedure was 123,344 ± 73,222 SEK (12,021 ± 7,512 EUR). In total, there were 506 procedures carried out under C15O at a mean cost of 22,276 ± 23,689 SEK (2171 ± 2309 EUR) per contact. The mean cost of patients requiring this procedure was 70,778 ± 60,322 SEK (6898 ± 5879 EUR). To gain an understanding of how surgical interventions may influence healthcare utilization over time, the impact of surgeries on postoperative healthcare utilization in the high-cost cohort were also examined with time (Fig. 2). The mean costs during the year of first surgery were found to be 120,386 ± 73,539 SEK (11,733 ± 7167 EUR). On average, healthcare costs increased during the years following middle ear surgeries, from an average yearly cost of 11,594 SEK (1130 EUR) over the four years leading to surgery to 17,798 SEK (1926 EUR) for the proceeding four years. Because ossicular reconstructions were captured under a unique surgical code we were able to investigate these procedures separately. For patients that underwent ossiculoplasty procedures, a similar pattern of healthcare utilization was observed to those placed in the high-cost quartile (Fig. 3). Average healthcare costs increased during the years following ossiculoplasty procedures for these patients, from an average yearly cost of 9820 SEK (957 EUR) over the four years leading to surgery to 17,273 SEK (1684 EUR) for the proceeding four years.

## Discussion

There are major deficiencies in the knowledge surrounding healthcare utilization, including audiological care, in patients with chronic otitis media. Registry studies provide insights into real-world outcomes for large populations that can be used to guide decisions surrounding resource utilization to the benefit of patients and payers. This is of particular importance in countries where societal resources are limited. The strengths of the study include the large number of patients with otitis media and a design that permits the reporting of the normal care pathway for these patients, including both primary and secondary care. However, it should be noted that these data are collected from a Swedish patient register and will not be directly transferrable to other healthcare systems. A previous study has investigated healthcare utilization and prescribing patterns in patients with eustachian tube dysfunction [18] and, at the time of writing, a retrospective study investigating healthcare utilization in the USA also documented the high healthcare utilization and associated costs in patients with chronic otitis media [19].

The present study also supports the finding that chronic otitis media is associated with a high level of health care utilization. The data show that most patients make multiple contacts with health care providers each year and that this does not decrease over time. Chronic conditions are typically associated with high resource utilization, making them a target for tailored interventions to reduce resource wastage [20]. One explanation for high resource utilization in our dataset may be due to the fact that the effectiveness of surgeries that aim to restore hearing diminish over time and revision surgeries are necessary to maintain satisfactory hearing outcomes [21, 22]. Revision surgeries were recorded for 25% of patients in the costliest quartile and 19% of all patients that underwent an ossiculoplasty required a revision ossiculoplasty. These data indicate that revision surgeries, including those focused on hearing rehabilitation, are drivers of healthcare costs for this patient group. On average, the most expensive quartile of patients also made ten additional visits to their healthcare provider over five years. It should be noted that the time between primary and revision tympanoplasty can range between 10 and 23 months [23] and the mean time between primary and revision ossiculoplasty has been reported to be 36 months [24], we are therefore most likely to capture one revision surgery during our review period in a situation where some patients may require several revision surgeries to reach an acceptable audiological outcome [25]. Cost–utility analysis for tympanomastoid surgery in 77 adults with chronic suppurative otitis media has been undertaken in Taiwan [26]. The study captured direct healthcare costs during the first postoperative year only but, in line with our study, also found that the highest proportion of costs (51.8%) were due to operation-related fees. Their model identified that treating patients with active aural discharge was most cost-effective, but the authors affirm that better disease subclassification and outcomes data were still required to build a robust, cost-effectiveness model for tympanomastoid surgeries. Based on the reported costs associated with ossiculoplasty procedures, it is motivating to assess how this might compare to alternative forms of hearing rehabilitation to treat these patients, such as conventional hearing aids, bone conduction devices, or cochlear implants. Future studies should better characterize the cost-effectiveness of these interventions as patients with COM are known to undergo repeated surgeries while at the same time carrying the burden of hearing loss [27]. In an attempt to find sustainable solutions for these patients, it will be important to quantify the problem and, if possible, identify prognostic factors that may guide more effective hearing rehabilitation. Future clinical studies should be planned to determine whether alternative solutions to current practice could offer a more effective and cost-effective rehabilitation and hearing rehabilitation in patients with COM. These data are not obtainable from registry studies alone since they cannot provide complete information covering diagnostics, co-morbidities and patient care. Furthermore, as care providers, especially those in primary care, do not always specify a diagnosis when registering care contacts, some healthcare contacts may go unrecorded, leading to an underestimation of healthcare consumption. Coding practices can also differ among clinics and specialties leading to under or overestimations of healthcare utilization. A key limitation of the current study is that similar codes are aggregated in the registry output, which prevents specific procedures from being attributed to individual subjects. This inherent shortcoming makes it difficult to draw conclusions on unilateral versus bilateral cases and on subjects with and without cholesteatoma, two important factors which will likely have a large impact on healthcare utilization. Studies of other designs, such as retrospective chart reviews or randomized controlled trials, will be necessary to supplement registry data to drive a change in treatment practice.

## Conclusion

In Sweden, COM-related healthcare utilization and costs are substantial and a large proportion of costs are attributed to revision tympanoplasties. This study represents a next step towards optimal care of patients with COM. Optimized treatment will eventually reduce healthcare costs and improve hearing and health-related quality of life in this patient population.
